# SLE and multiple myeloma: an underlooked link? A review of case reports from the last decade

**DOI:** 10.25122/jml-2023-0314

**Published:** 2024-02

**Authors:** Rupak Desai, Sanjana Devaragudi, Loveneet Kaur, Kulwinder Singh, Jerrin Bawa, Nyein Wint Yee Theik, Spandana Palisetti, Akhil Jain

**Affiliations:** 1 Independent Researcher, Atlanta, GA, USA; 2 Department of Medicine, Apollo Institute of Medical Sciences and Research, Hyderabad, India; 3 Department of Medicine, Government Medical College, Patiala, India; 4 Department of Internal Medicine, Flushing Hospital Medical Center, NY, USA; 5 Department of Internal Medicine, Memorial Healthcare System, Hollywood, FL, USA; 6 Department of Medicine, Jawaharlal Nehru Medical College, Belagavi, Karnataka, India; 7 Department of Leukemia, The University of Texas MD Anderson Cancer Center, Houston, TX, USA

**Keywords:** myeloma, SLE, lupus, multiple myeloma

## Abstract

Systemic lupus erythematosus (SLE) affects multiple organ systems, and there has recently been increasing evidence that suggests a considerable rise in cancer risk. Despite growing evidence, the relationship between SLE and multiple myeloma (MM) remains underlooked. This review synthesizes findings from case reports published between 2012 and 2023 to explore this relationship. We conducted a comprehensive search using PubMed, Embase, and Google Scholar with the keywords 'SLE' and 'multiple myeloma' and described the clinical profile of MM in patients with SLE. Seven case reports were reviewed. Five case reports included female participants, two had a simultaneous diagnosis of SLE and MM, and in others, MM followed SLE varying from 7 months to 30 years. Two cases reported an improvement in MM. Four cases reported death due to complications, which included shock, myocardial infarction, and pneumonia. Lupus nephritis was seen to complicate MM and SLE complex in 2 cases. Larger, well-developed studies focusing on clinical presentation, diagnostic strategy, treatment, and outcomes are needed to better understand the association between SLE and MM. Healthcare workers should be aware of the increased risk of malignancy in SLE and customize screening accordingly.

## INTRODUCTION

Systemic lupus erythematosus (SLE) is among the most prevalent systemic autoimmune disorders, with significant incidence and prevalence observed in North America and among African American populations. It predominantly affects women and often manifests with a peak incidence in middle age [1]. SLE is known to affect multiple organ systems, but there has recently been increasing evidence suggesting a considerable rise in cancer risk compared to the normal population. This includes both solid and hematologic malignancies [2]. Among these, multiple myeloma (MM) presents the most significant risk, with a standardized incidence ratio (SIR) of 8.9 (95% CI, 3.90–12.48) [3]. A meta-analysis from 2014 also identified a four-fold increased risk of MM (95% CI, 1.98–8.87) in SLE [4]. The pathogenesis associated with this increased risk is not clearly understood [2,5]. The link remains underlooked despite advances in understanding the pathobiological changes between immunological and malignant diseases, highlighting the pressing need to study the cases of SLE leading to MM. Therefore, to address this knowledge gap and improve patient care, we conducted a review to describe the patient demographics, presentations, co-morbidities, treatment approaches, and outcomes of MM in patients with SLE. By providing valuable clinical data on this specific patient population, this review can raise awareness among healthcare professionals about the potential for MM development in SLE, ultimately facilitating earlier diagnosis and treatment interventions.

## MATERIAL AND METHODS

**Figure 1:**
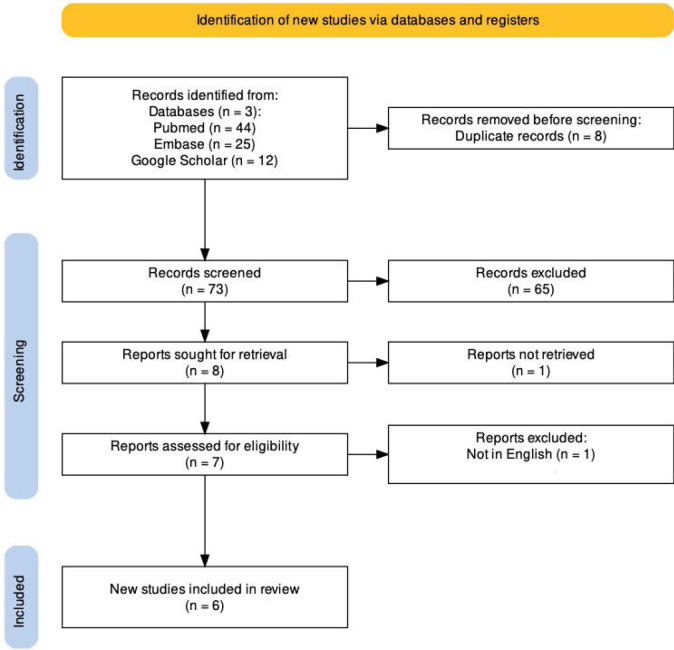
PRISMA flowchart

The review was conducted in accordance with the Preferred Reporting Items for Systematic Review and Meta-Analysis (PRISMA) guidelines (Figure 1.) We conducted a systematic literature search across PubMed, Embase, and Google Scholar for case reports published from January 1^st^, 2012, to February 1^st^, 2023, using the combined keywords ‘systemic lupus erythematosus’ AND ‘multiple myeloma’. We included case reports involving adult patients (18 years and above) with confirmed diagnoses of SLE and MM. We excluded studies involving only SLE or MM diagnosis, published in languages other than English, and studies involving participants in the pediatric age group.

The title and abstract screening were done by three independent authors according to the inclusion and exclusion criteria. Duplicates were removed. Subsequently, two reviewers read the full text to confirm that the articles met the criteria. Any conflicts were resolved by a third author. The quality assessment of studies was done by two independent authors using the Joanna Briggs Institute (JBI) critical appraisal tool, which is a recommended tool for case reports. Eight indicators were used. The data from each study were extracted and added to a table in Microsoft Excel. Three independent authors completed the data extraction, which was reviewed by another independent author. The extracted data from the included cases are summarized and reported in Table 1.

**Table 1:** Systemic Lupus Erythematosus associated Multiple Myeloma – Published Case Reports (2012-2023)

Author	Age	Sex	Comorbidities	Presenting symptoms	Age at SLE diagnosis	Treatment for SLE	Hb	Sr. Calcium	Sr. Creatinine	BUN	Urine protein on U/A	24 hr urinary protein	Serum Electrophoresis	24 hours Bence Jones Poteins	Bone marrow aspirate/ biopsy	Treatment for MM	Outcome	Complications
Lian *et al*. Case 1	59	M	N/A	Joint swelling and pain, fever	48	Prednisone acetate 60mg - 5mg, hydroxychloroquine 200mg, cyclosporine 150mg (Stopped 4 years back)	13.4 g/dL	1.89 mmol/L	2.6 mg/dL	42.21 mg/dL	Negative	270 mg/ 24hours	IgG λ with free light chains	N/A	Revealed multiple myeloma	Three cycles of bortezomib, dexamethasone, and chemotherapy with liposomal doxorubicin. One cycle of lenalidomide and dexamethasone	Death	Infectious shock
Lian *et al*. Case 2	58	F	N/A	Blood pressure of 200/100 mm of Hg, anemic appearance, mild edema of lower limbs	31	Cyclophosphamide 60mg - 10mg, mycophenolate mofetil 0.75 g, hydroxychloroquine 300mg, prednisone acetate 10 mg	9.3 g/dL	3.5 mmol/L	8.7 mg/dL	95.6 mg/dL	2+	1.43 g/ 24hours	IgG γ with free light chains	N/A	N/A	N/A	Death	Myocardial infraction
Hamza *et al*.	57	N/A	Hypertension	Microscopic hematuria	27	Tacrolimus 4 mg, prednisolone 15 mg, Vitamin D3 1000 unit capsule, calcium carbonate 1500 mg, Vitamin B complex one tablet.	9.4 g/dL	N/A	1.23 mg/dL	19 mg/dL	15 mg/dL	898 mg/ 24hours	Elevated gamma globulin with a spike in the gamma region (IgG 3.1 gm/dl)	Free kappa light chain positive	Hypercellular marrow showing aggregates of closely packed monoclonal plasma cells comprising more than 15% of the marrow cellularity	N/A	N/A	Class IIa lupus nephritis, associated with light chain induced proximal tubulopathy
Chen *et al*.	69	F	Hypertension, hyperlipidemia, breast cancer	Fever, rhinorrhea, hoarseness, itching skin, and rashes over her trunk and limbs	69	Intravenous hydrocortisone 50mg, hydroxychloroquine 200mg	8.1 g/dL	2.07 mmol/L	1.1 mg/dL	18 mg/dL	300 mg/dL	4400 mg/ 24hours	Increased IgG >3000mg/dL	Free kappa light chain = 53437.5 mg/L; free lambda light chain = 345 mg/L	Plasma cells exceeded 10% of the marrow nucleated cells	One capsule of thalidomide	Death	Multiple organ failure caused by pneumonia and respiratory failure
Humayun *et al*.	35	F	N/A	High grade fever,shortness of breath,joint pains,backache,lethargy,undocumented weight loss	35	Prednisolone 45mg/day,Hydroxychloroquine 400 mg/day,mycophenolate mofetil 1g/day	6.5 g/dL	Normal	Normal	Normal	Albumin ++, 20-25 pus cells	1.2g/ 24hours	Elevated gamma globulins 57.5% mainly IgG type	Negative	30 % Plasma proteins	Melphalan at dose of 8mg/day for four days to be repeated every 7th week	Improved status	Lupus nephritis
Wang *et al*.	47	F	Systemic sclerosis	Lumbar pain,Chest pain (pain in ribs exacerbated with movement)	41	Prednisone 30mg daily,hydroxychloroquine 400mg daily,cyclophosphamide 800mg monthly	6.2 g/dL	N/A	Normal	Normal	RBCs 45000/mL with 65% being heterotypic	1.788g/ 24 hours	Kappa type M protein	Free kappa light chain positive	Decrease in marrow hyperplasia with rouleau formation,normal morphology of RBC along with plasmacytosis (48%)	Chemotherapy with vincristin,doxorubicin,dexamethasone	Death	Severe Pneumonia
Ding *et al*.	48	M	N/A	Recurrent pain of multiple joints (Bilateral shoulders,elbows,knees,metacarpophalengeal joint,PIP’s),one hour’s morning stiffness of B/L hands,fever	45	IV methylprednisolone 40mg/day,methotrexate 10mg/week	11.3 g/dL	N/A	Normal	Normal	N/A	56mg/ 24 hours	Monoclonal immunoglobulin of IgG/λ	N/A	BM aspirate- increased plasma cells of which 14% nucleated cells & 3% immature plasma cells. Biopsy- increased plasma cells disseminated in bone marrow which suggested plasma cell tumor	Six cycles of CTD regimen chemotherapy (cyclophosphamide 0.4 g on day 1 to 4, dexamethasone 40 mg day 1 to 4, thalidomide 100 mg orally every night. Autologous peripheral blood stem cell transplantation	Improved	N/A

## RESULTS

This review identified six case reports describing seven patients with co-existing SLE and MM.

**Case 1:** A 59-year-old man diagnosed with SLE at 48 years old presented with fever, joint discomfort, and edema. He was treated with prednisone acetate, hydroxychloroquine, and cyclosporine. Following an MM diagnosis, he received bortezomib, dexamethasone, chemotherapy using liposomal doxorubicin, and lenalidomide. He died due to septic shock [6].

**Case 2:** A 58-year-old woman diagnosed with SLE at 31 years old presented with high blood pressure, an anemic look, and slight lower limb edema. Her SLE treatment included prednisone acetate, hydroxychloroquine, mycophenolate mofetil, and cyclophosphamide. She did not receive any treatment for MM and died due to myocardial infarction [6].

**Case 3:** A 57-year-old woman with hypertension, diagnosed with SLE at 27 years old, presented with microscopic hematuria. Her initial SLE treatment involved tacrolimus, prednisone, vitamin D3, calcium carbonate, and vitamin B complex. Further evaluation revealed class IIa lupus nephritis linked to light chain-induced proximal tubulopathy and a diagnosis of MM. Treatment was not specified [7].

**Case 4:** A 69-year-old woman with a history of treated breast cancer, hypertension, and hyperlipidemia presented with fever, rhinorrhea, hoarseness, itchy skin, and rashes on her trunk and limbs. She was diagnosed with SLE and MM simultaneously. Hydroxychloroquine and intravenous hydrocortisone were used to treat SLE, and one thalidomide pill for MM. She died due to multiple organ failure caused by pneumonia and respiratory failure [8].

**Case 5:** A 35-year-old woman presented with high-grade fever, dyspnea, joint and back discomfort, tiredness, and an unrecorded weight loss. She was simultaneously diagnosed with SLE and MM and treated with prednisolone, hydroxychloroquine, and mycophenolate mofetil. Melphalan was used to treat MM after her SLE improved [9].

**Case 6:** A 47-year-old woman diagnosed with SLE and systemic sclerosis at 41 years old presented with chest discomfort and lumbar pain. Her initial SLE treatment included prednisone, hydroxychloroquine, and cyclophosphamide. Upon her MM diagnosis, she received vincristine, doxorubicin, and dexamethasone. She died due to severe pneumonia [10].

**Case 7:** A 48-year-old man with frequent joint discomfort, morning stiffness, and fever was diagnosed with SLE and treated with intravenous (IV) methylprednisolone and methotrexate. After 7 months, he was diagnosed with MM. He underwent six cycles of chemotherapy using the CTD regimen (cyclophosphamide, thalidomide, and dexamethasone). Autologous peripheral blood stem cell transplantation was done with improvement in SLE and MM [5].

## DISCUSSION

SLE and MM are complex autoimmune and neoplastic disorders, respectively, affecting multiple organ systems. Diagnosing and treating them individually is challenging, and both conditions in a single patient present even greater difficulties. The overlapping manifestations, like pancytopenia and renal function impairment, often lead to a missed or late diagnosis [6,8]. Lian *et al*. [6] described a case with renal impairment and progressively increasing serum creatinine levels for four years before diagnosing MM and thereby recommended the importance of screening patients with SLE for MM. On the other hand, few clinical features like lytic bone lesions or plasmacytoma favor MM over SLE.

Five of the seven cases we reviewed included female patients. A study revealed that women with SLE have a higher risk of MM (SIR = 8.95, 95% CI, 4.08–13.82) compared to men (SIR = 3.90, 95% CI, 0–11.53) [3]. The lack of standardized guidelines underscores the need for further research to develop optimal treatment strategies for this concurrent disease complex. Currently, physicians manage MM in patients with SLE on a case-by-case basis, integrating existing SLE maintenance with their preferred MM regimen [9]. Common treatment regimens included VAD (vincristine, adriamycin, and dexamethasone) and MP (melphalan and prednisolone) [5,6,9]. Thalidomide was sometimes used as a second-line drug [9]. Other drugs mentioned in this review were CTD regimens (cyclophosphamide, thalidomide, and dexamethasone), bortezomib, dexamethasone, and doxorubicin [5,6]. Autologous peripheral blood stem cell transplantation was described in one case [5]. The outcomes and complications described among cases varied. Two cases reported an improvement in MM [5,9]. Four cases reported death due to complications, which included shock, myocardial infarction, and pneumonia. Lupus nephritis was seen to complicate MM and SLE complex in two cases [7,9].

The exact mechanisms behind concomitant SLE and MM pathogenesis are not fully understood. Steroids have a well-established role as the primary drug for both SLE and MM treatment, indicating an underexplored and unclear immunological common pathway. Goobie *et al*. [11] reviewed various possible hypotheses for different malignancies in SLE with an emphasis on hematological malignancies. Autoantibodies in rheumatological diseases trigger dysregulation of B and T cells and their subsequent function, allowing the carcinogenic protein to proliferate unchecked. SLE therapies that include immunosuppressants modify signaling pathways, cytokines (e.g., interleukin-6), and tissue milieu and induce de-novo carcinogenic genetic lesions.

## CONCLUSION

Due to the rarity of cases involving SLE and MM, prevalence data are scarce. However, this could be due to a missed diagnosis. Larger, well-developed studies focusing on clinical presentation, diagnostic strategy, treatment, and outcomes are needed to investigate the association between SLE and MM. Such research would inform the development of guidelines for timely screening, appropriate diagnostic approaches, and effective management protocols for MM in patients with SLE. Additionally, a better understanding of the prognosis of this disease complex could be achieved. Our study highlights the importance of early detection, management of comorbidities, and increased surveillance strategies for SLE patients. Healthcare policies and guidelines should consider incorporating recommendations for cancer screening tailored to SLE, especially for MM.
